# Supplementary material for: From sequence to enzyme mechanism using multi-label machine learning
Source: BMC Bioinformatics. 2014 May 19;15:150. doi: 10.1186/1471-2105-15-150 (PMC4229970; doi:10.1186/1471-2105-15-150)
Supplement: Additional file 2 — Java code of ml2db. Additional file ml2db_code.tar.gz contains the Java source code to run the multi-label machine learning experiments and save the results to database. The code’s Javadoc is included. [file 1471-2105-15-150-S2.zip › additional file 2/ml2db/ml2db/doc/test/ml2db/dataharness/package-summary.html]

test.ml2db.dataharness


JavaScript is disabled on your browser.


- Overview
- Package
- Class
- Use
- Tree
- Deprecated
- Index
- Help

- Prev Package
- Next Package

- Frames
- No Frames

- All Classes

# Package test.ml2db.dataharness

- Class Summary

  | Class | Description |
  | --- | --- |
  |  |  |
  | --- | --- |
  | ArffPropsFilesTest | Test the ARFF properties files used in the test harness and their content. |
  | ArffPropsOneTest | Get the arff data properties from file |
  | ArffPropsQueriesOneTest | Class |
  | ArffPropsQueriesTest | Checks the results of the queries contained in the arff properties files |
  | ArffPropsQueriesTwoTest | Class |
  | ArffPropsTwoTest | Class |
  | DatabaseTest | Check the test database |
  | DataOne | Class |
  | DataTableFourTest |  |
  | DataTableOneTest | Basic data table containing 3 columns with: instances, attributes, classes. |
  | DataTableTest | Create a table for test instances (and their attributes and classes) |
  | DataTableThreeTest |  |
  | DataTableTwoTest |  |
  | DataTwo | Class |
  | LoadFirstValidArff | Class |
  | TestProjectParameters |  |

- Overview
- Package
- Class
- Use
- Tree
- Deprecated
- Index
- Help

- Prev Package
- Next Package

- Frames
- No Frames

- All Classes
